# Supplementary material for: Switching speed limits in electrically driven VO2 structural Mott–Peierls transition
Source: Nat Commun. 2026 Feb 24;17:3139. doi: 10.1038/s41467-026-69904-0 (PMC13044288; doi:10.1038/s41467-026-69904-0)
Supplement: Supplementary file 1 — Supplementary Information [file 41467_2026_69904_MOESM1_ESM.pdf]

Supplementary Information

Switching Speed Limits in Electrically Driven VO_2 Structural Mott–Peierls Transition

A. Pofelski[1], C. Liu[1], S.A. Reisbick[1], MG Han[1], L. Wu[1], H. Navarro[2,3], E. Qiu[2], T. D. Wang[2], S. Mousavi[4], D.J. Alspaugh[2], M. Rozenberg[2,5], S. Ramanathan[6], I.K. Schuller[2], and Y. Zhu[1]

[1] Condensed Matter Physics and Materials Science Department, Brookhaven National Laboratory, Upton, New York 11973, United States
[2] Department of Physics, University of California San Diego, La Jolla, California 92093, United States
[3] Department of Physics, Andrews University, Berrien Springs, Michigan 49104, United States
[4] Clean Energy Innovation Research Centre (CEI), National Research Council Canada Mississauga, ON L5K 1B4, Canada
[5] CNRS Laboratoire de Physique des Solides, Université Paris-Saclay, 91405 Orsay, France
[6] Department of Electrical and Computer Engineering, Rutgers, The State University of New Jersey, Piscataway, New Jersey 08901, United States

Figures

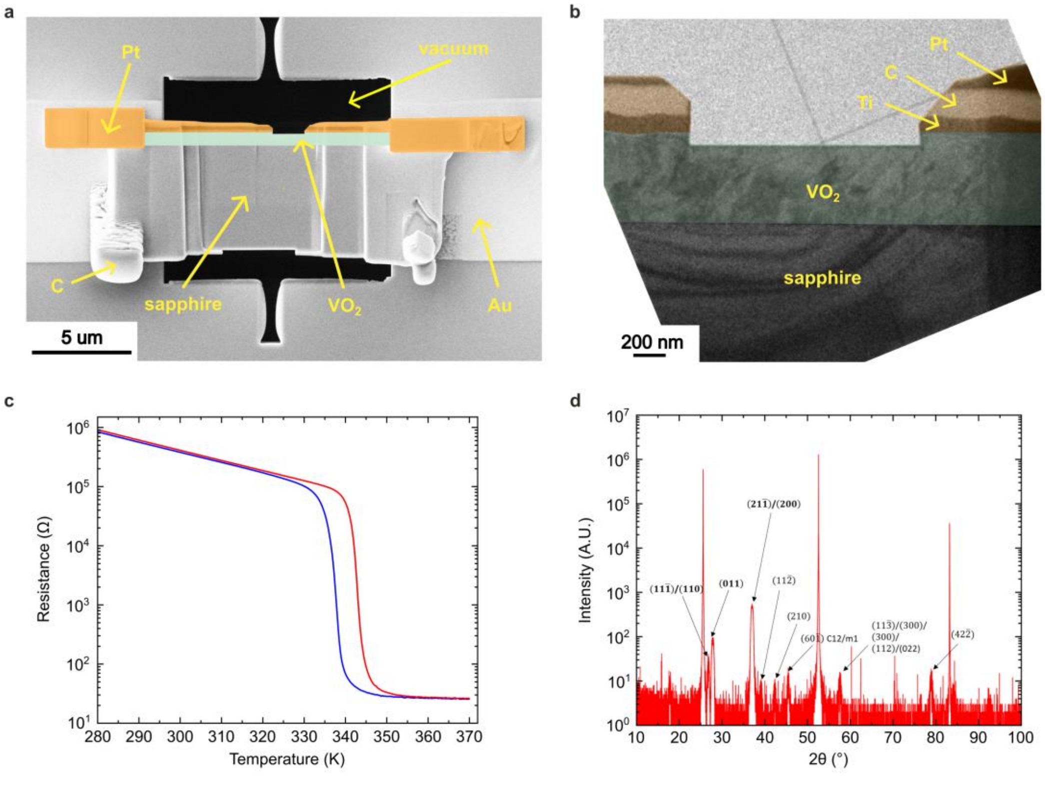

Figure S1: a, Scanning Electron Microscopy image of the VO_2 lamella positioned above the open window and connected laterally with Pt to the Au lines from the custom chip. b, Transmission

Electron Microscopy image of the VO_2 two-terminal device. c, Resistance vs temperature measurement of the VO_2 thin film on sapphire substrate. d, X-Ray Diffraction measurement of the same VO_2 thin film on sapphire.

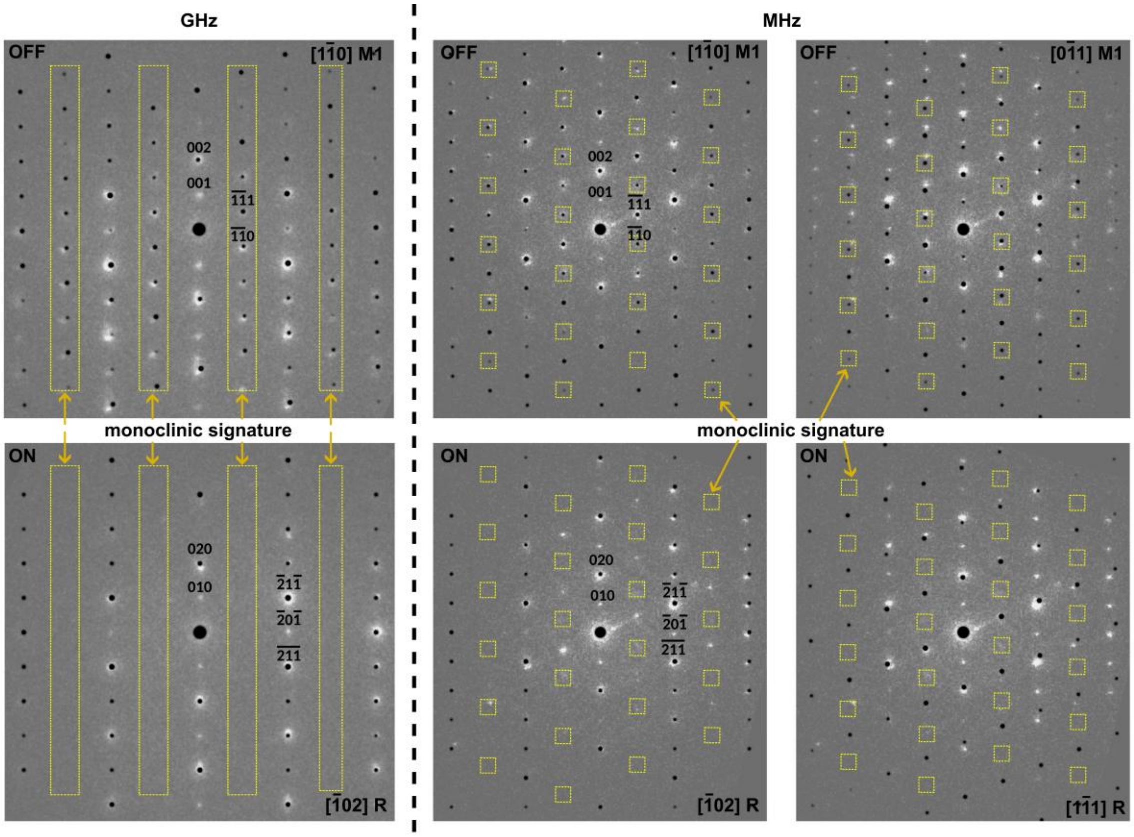

Figure S2: Indexing and localization of the monoclinic diffraction peaks from the Selected Area Electron Diffraction patterns recorded in the GHz and MHz setup. The MHz diffraction pattern is an overlap of two grains with different orientations and sharing common diffraction peaks.

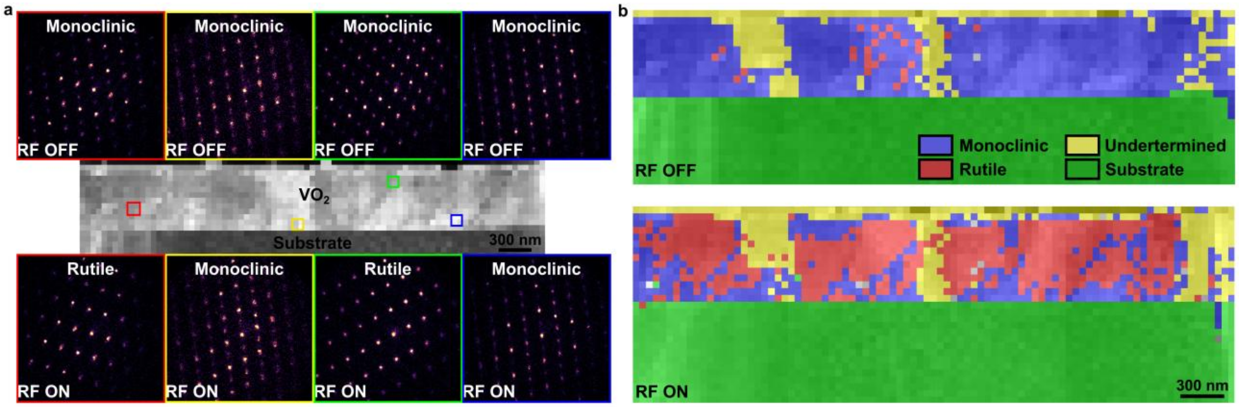

Figure S3: In-situ RF characterization of the VO_2 thin film grown on sapphire. a, 4D-STEM map under RF excitation and several averaged electron diffraction patterns from the total intensity map. b, Phase map distribution of the monoclinic and rutile domains with RF off and on. A machine learning-assisted method is employed for detailed, pixel-by-pixel visualization of the phase map. Specifically, a small convolutional neural network is trained on a total of 480 diffraction patterns (2816 diffraction patterns after augmentation). These patterns include 406 expertly labeled phase diffractions and 74 simulated datasets (simulations done using the py4dstem python package[1]), each deliberately altered with artifacts sch as rotation, noise, and shift. The training set is balanced to prevent class biases. The convolutional network was designed not for generalization but to facilitate the classification and visualization of diffraction maps, guided by expert labels and simulations. The training accuracy achieved was 88.3 %, and results have been double validated by experts. The saved models, training outcomes and raw 4D-STEM data are available in the dedicated repository[2] (https://doi.org/10.5281/zenodo.14767722).

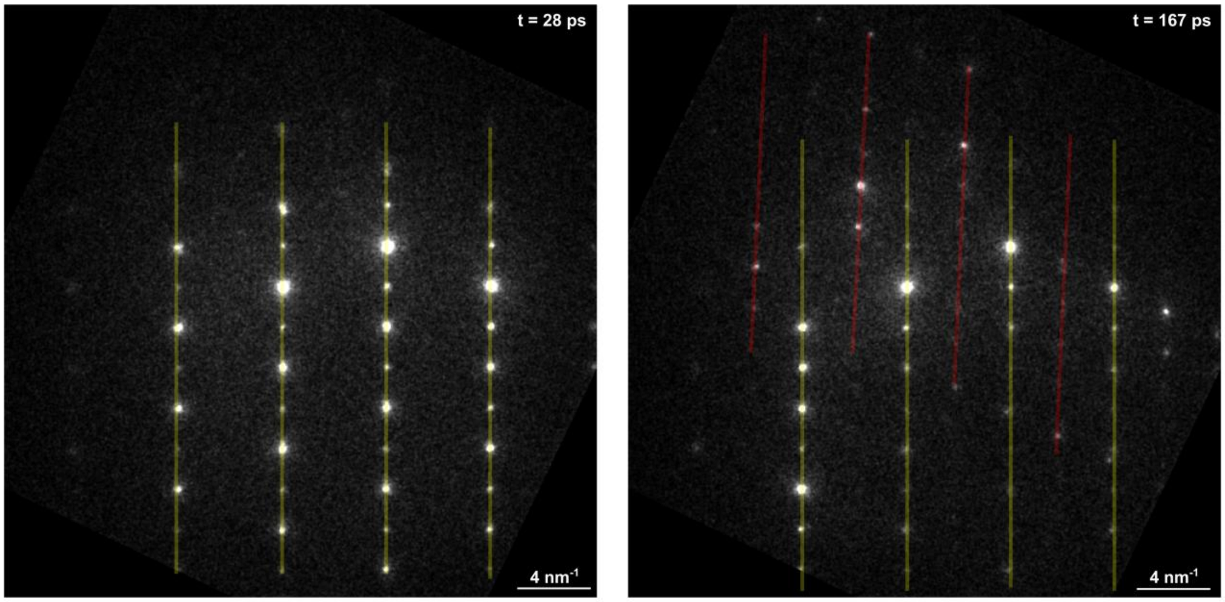

Figure S4: Ultrafast Selected Area Diffraction from supplementary movie 2 at two different time points. The diffraction peaks under the yellow lines correspond to one grain (grain of interest), and the diffraction peaks under the red lines correspond to another grain near the substrate. The change in the diffraction peaks arrangement is due to sample drift during RF phase shifter slightly changing impedance when shifting the phase as shown in supplementary movie 8.

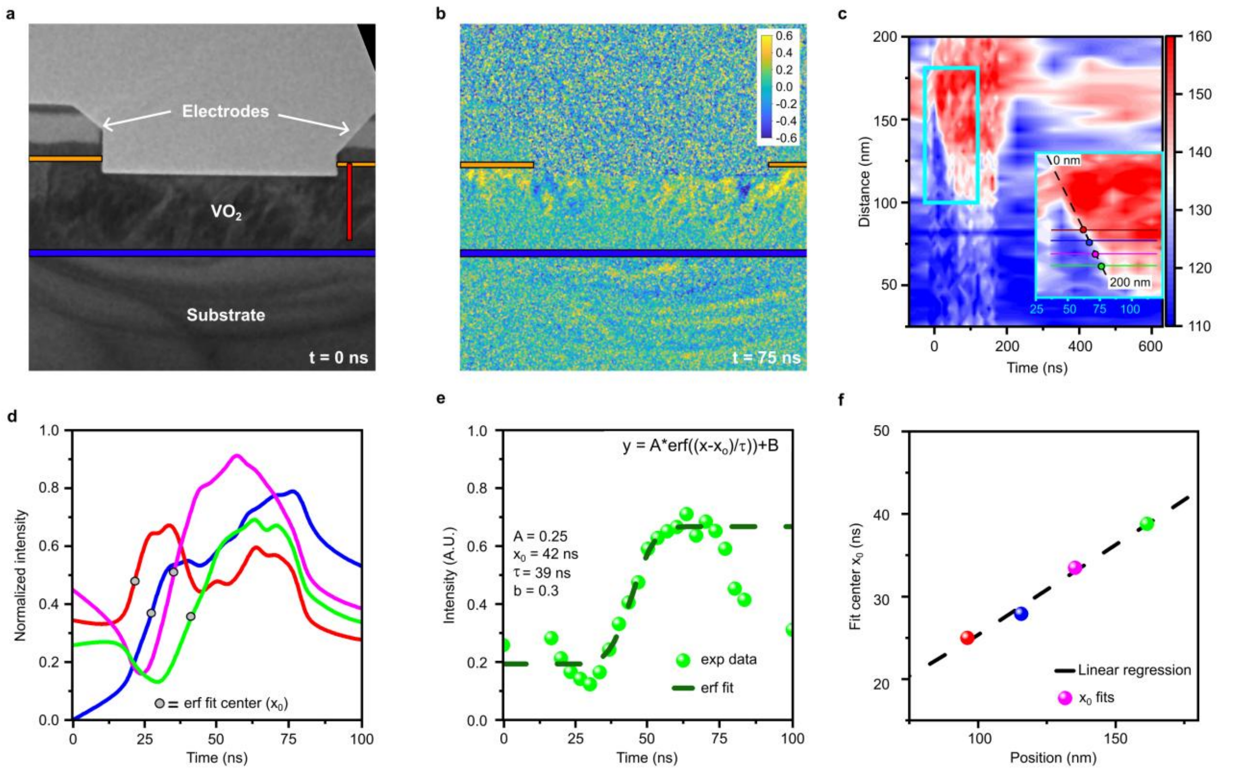

Figure S5: Extraction of the time constant and the structural phase front velocities. a, Bright-field UTEM image with the VO_2 sample, substrate and electrodes highlighted. b, Difference between two normalized images at t=75 ns and t=0 ns. Line annotations are used to distinguish the sample, substrate and electrodes. c, Space time contour plot (STCP) of along the red line in panel a. Each column corresponds to the intensity line profile starting from the bottom to the top of the red line in a, for each image in the time sequence over a cycle. The inset contains the segment of the image sequence corresponding to the growth of the rutile phase downwards the VO_2 film. d, Horizontal line profiles along the color-coded positions in panel c. The rise of each curve was fitted to an erf function (shown in panel e). e, Example of the fitting parameters used to extract the position of the rising edge of the rutile domain growth. F, Linear fit of the extracted time x_0 when the phase transition occurs with respect to the position in the STCP. The measurements demonstrate that the rutile phase grows from the electrode interface towards the substrate at a rate of 4.59 nm/ns. The difference in velocity in the manuscript arises from the transposed axes when fitting is performed, and the difference is proportional to the R^2 such that a value of 1 would have identical velocities after the transpose. Here, R^2 = 0.9892, so the velocity measurement differs by about 1%.

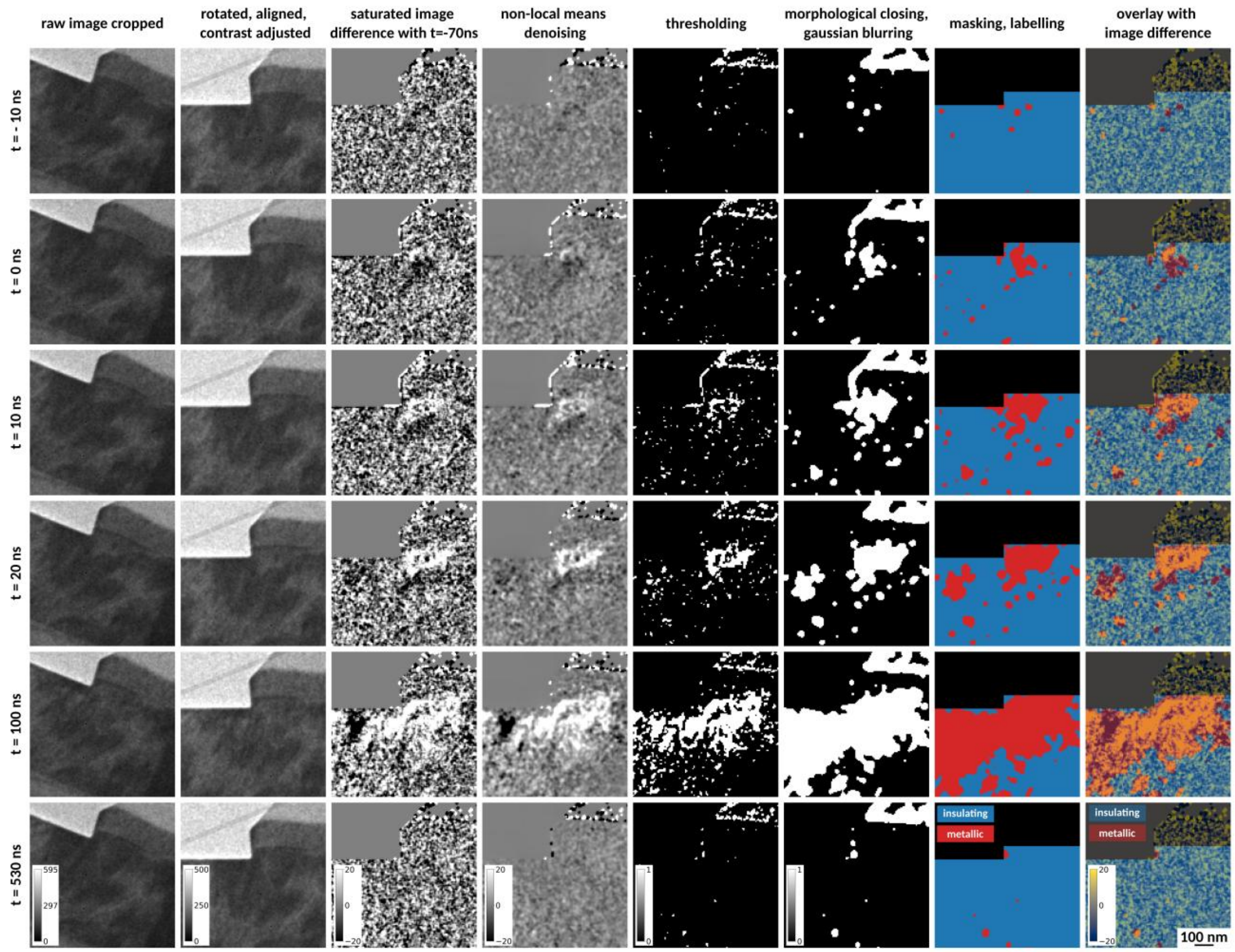

Figure S6: Overview of the image processing workflow of the ultrafast bright-field U(BF)TEM image series focusing on the right-hand side nucleation site of the VO_2 device at different time points of the 1 us cycle. In the main manuscript, the U(BF)TEM time series were rotated and aligned with each other to remove sample drift (rigid registration). At each time point, the image difference with t=-70 ns time point was performed to capture the change of contrast. The resulting image difference was gently smoothed with a 2d gaussian function and slightly saturated as shown above in the third column. The time t=0 was chosen when the change of contrast went above the noise level at the nucleation site on the image difference. In the supplementary information, the image difference time series was further denoised, threshold and filtered to label each pixel as insulating or metallic and guide the data interpretation.

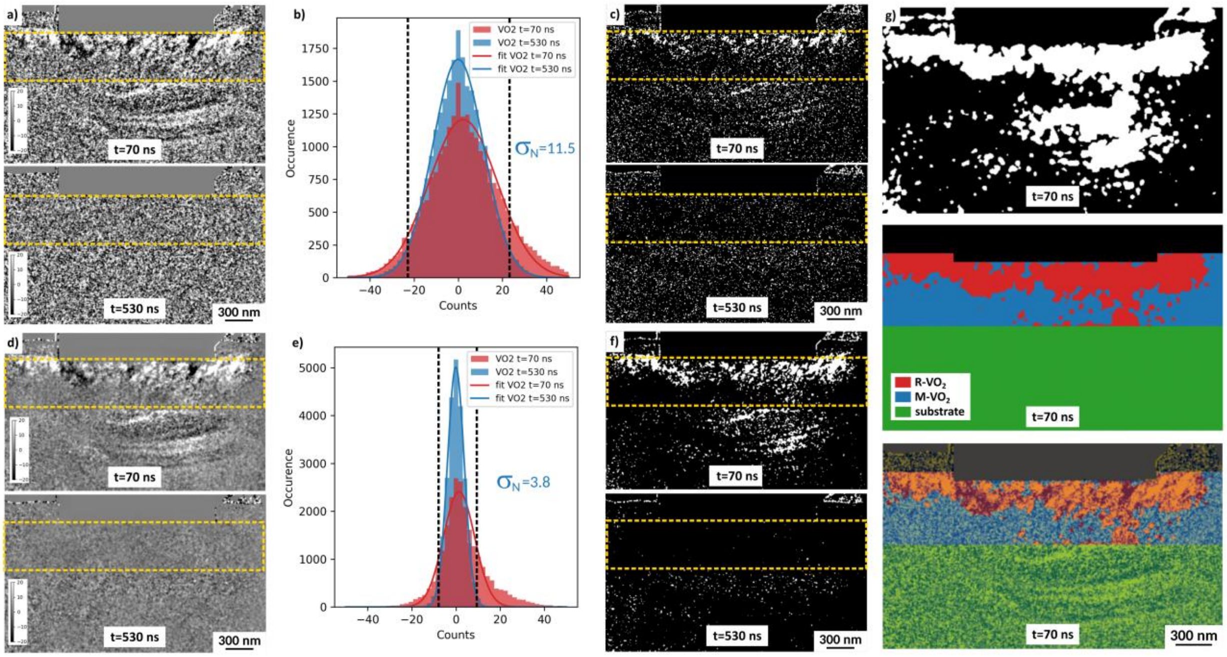

Figure S7: Segmentation of the rutile domains in the ultrafast bright-field (BF) TEM image difference at 1 MHz before and after non-local means denoising. a, Pulsed BF-TEM image difference at t=70 ns (filament formed), and t=530 ns (filament dissolved). b, Statistical distribution of the intensity from a) in the VO_2 region represented by the yellow dashed rectangle. c, Pulsed BF-TEM difference images from a), thresholded using a cutoff of +/- 23 (2*σ_N) as shown in b. d, Denoised pulsed BF-TEM image difference at t=70 ns and t=530 using non-local means algorithm. e, Statistical distribution of the intensity from d in the VO_2 region represented by the yellow dashed rectangle. f, Denoised pulsed BF-TEM difference images from d, thresholded using a cutoff of +/- 8 (2*σ_N) as shown in e. g, Morphological closing and Gaussian filtering of the image f, segmentation into rutile, monoclinic and substrate regions and overlay with the image pulsed BF-TEM image difference from a.

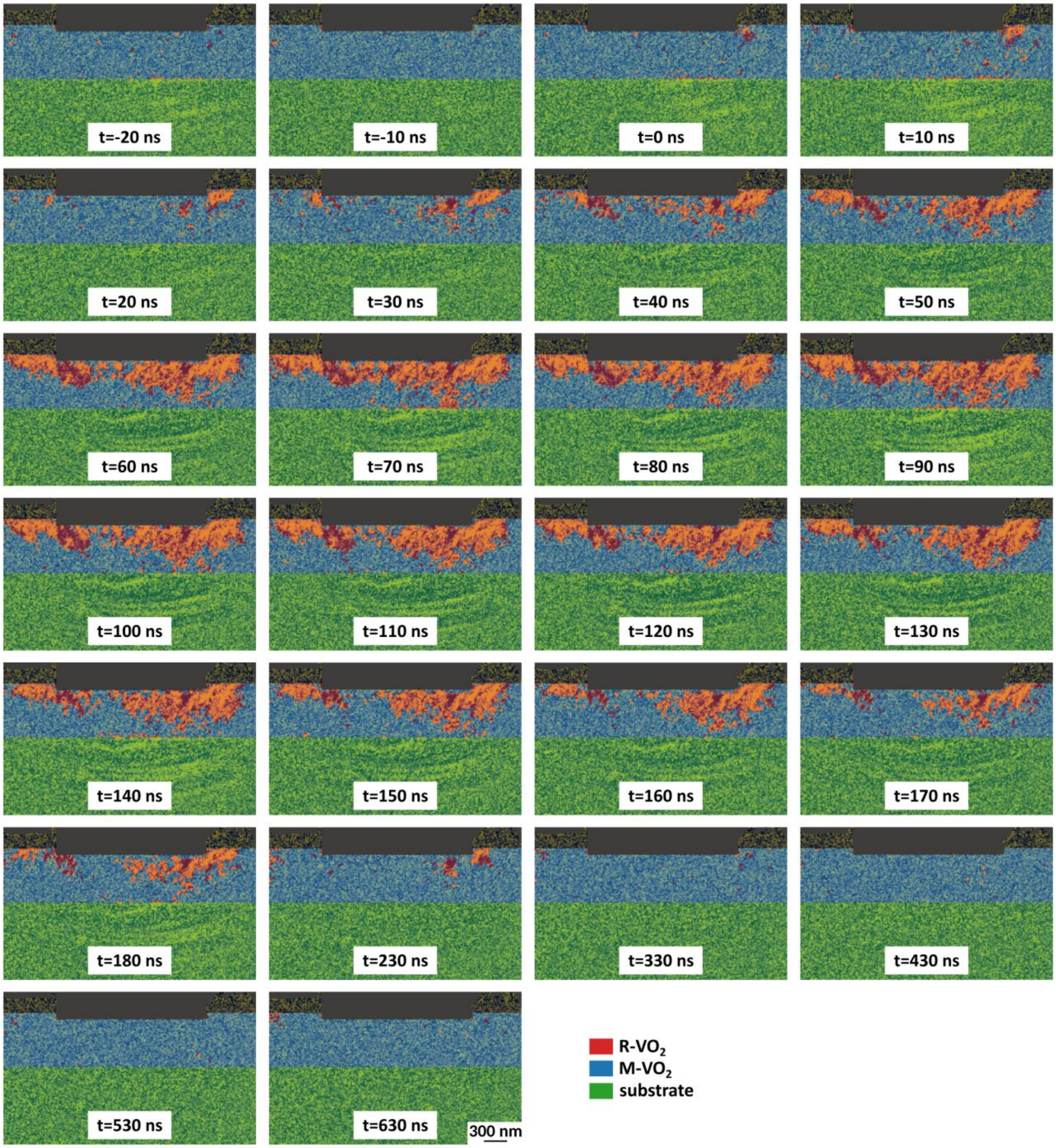

Figure S8: Phase mappings obtained using the methodology described in Fig. S6 and S7 overlayed with pulsed BF-TEM image difference at each time frame of the complete ultrafast experiment at 1 MHz electrical excitation and 90 ns pulse width.

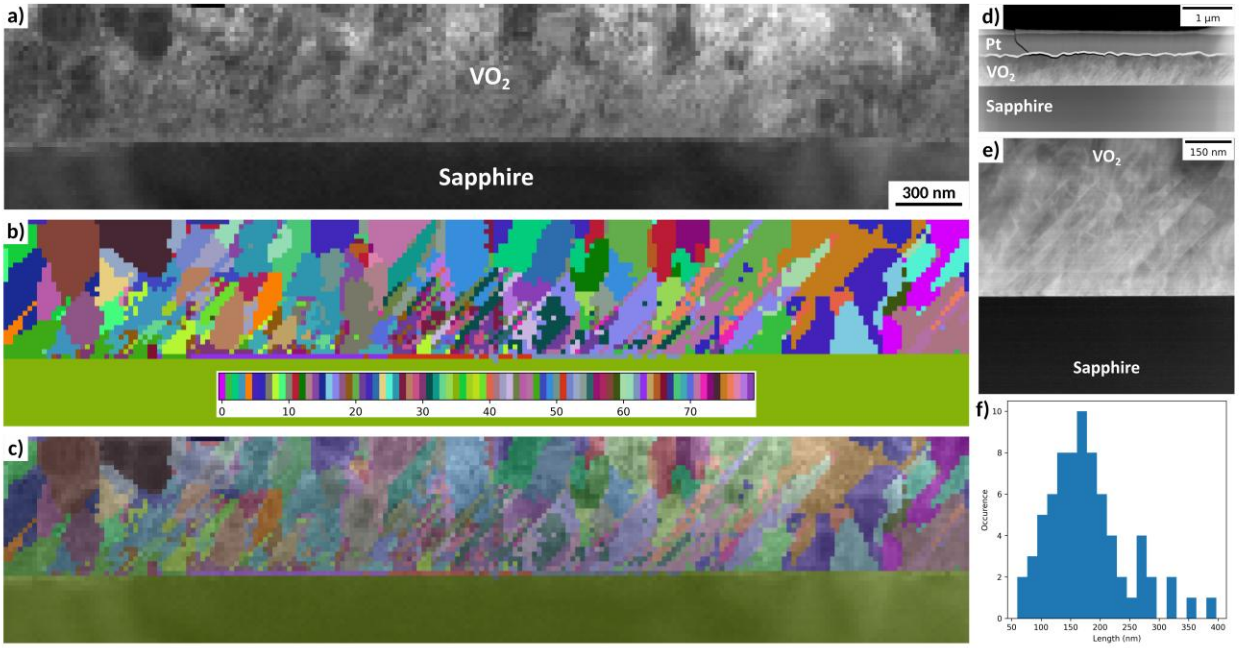

Figure S9: Qualitative overview of the VO_2 grain size distribution. a, Virtual bright field image from 4D-STEM mapping of the VO_2 film on sapphire. b, Cluster mapping regrouping regions with similar diffraction pattern using agglomerative hierarchal clustering. Each cluster represents qualitatively a VO_2 grain. c, Overlap of the cluster mapping b with the virtual BF image a. d, Large field of view STEM low angle annular dark-field image highlighting qualitatively the texture of the VO_2 film. e, STEM LAADF image on the bottom of the VO_2 film showing the inclination of small grains from the growth with sapphire. f, Statistical distribution of the grain size using the areas from cluster map converted into a length using the equivalent area diameter method.

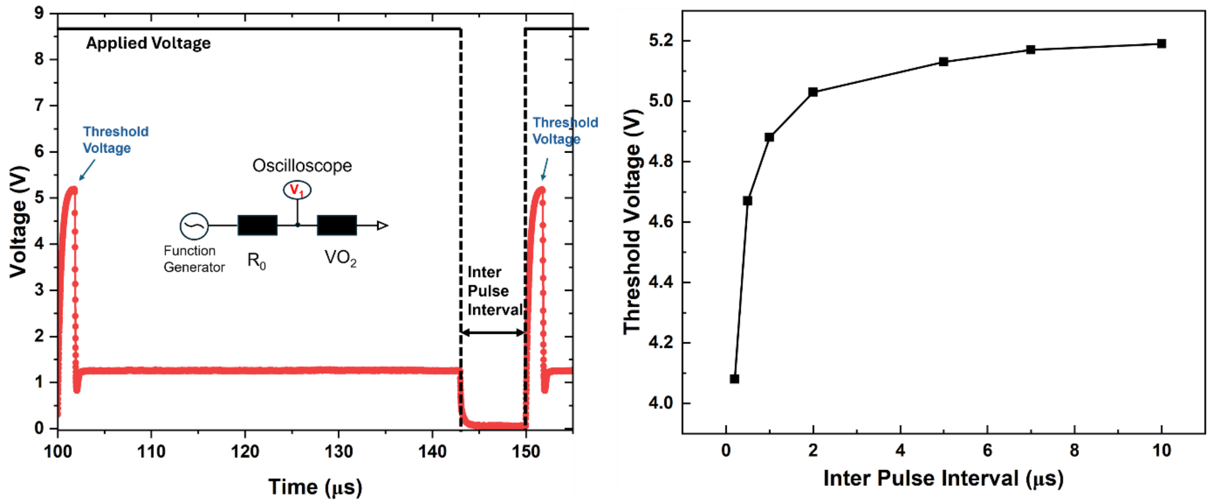

Figure S10: Time-resolved pulsed electrical measurement of a VO_2 device at 20 kHz. Left: Electrical measurement configuration showing the applied voltage pulse train (black dashed line) and the measured voltage response V1 across the device (red). Right: Extracted threshold voltage of the VO_2 device as a function of inter-pulse interval. For long inter-pulse intervals, the device fully relaxes to the high-resistivity monoclinic state, and the threshold voltage recovers to its nominal value. As the inter-pulse interval decreases, incomplete relaxation leads to a progressive reduction of the threshold voltage, indicating partial retention of the metallic state. Extrapolation toward shorter time intervals suggests that, at sufficiently high frequencies (above GHz), the threshold voltage effectively vanishes, leaving the device locked in a metallic state.

References

1. Savitzky, B. H., Zeltmann, S. E., Hughes, L. A., Brown, H. G., Zhao, S., Pelz, P. M., Pekin, T. C., Barnard, E. S., Donohue, J., Rangel DaCosta, L., Kennedy, E., Xie, Y., Janish, M. T., Schneider, M. M., Herring, P., Gopal, C., Anapolsky, A., Dhall, R., Bustillo, K. C., Ercius, P., Scott, M. C., Ciston, J., Minor, A. M., & Ophus, C, py4DSTEM: A Software Package for Four-Dimensional Scanning Transmission Electron Microscopy Data, *Microscopy and Microanalysis*, **27**, 4, 712-743 (2021), doi:10.1017/S143192762100047

2. Pofelski, A., Mousavi Masouleh, S. S. CNN_4DSTEM_VO2. *Zenodo* (2025), doi:10.5281/zenodo.14767723
