# Supplementary material for: Switching speed limits in electrically driven VO2 structural Mott–Peierls transition
Source: Nat Commun. 2026 Feb 24;17:3139. doi: 10.1038/s41467-026-69904-0 (PMC13044288; doi:10.1038/s41467-026-69904-0)
Supplement: Supplementary file 2 — Description of Additional Supplementary Files [file 41467_2026_69904_MOESM2_ESM.pdf]

Description of Additional Supplementary Files:

Supplementary Movie 1: Ultrafast Selected Area Electron Diffraction movie at 1 MHz electrical excitation with a pulse width of 90 ns.

Supplementary Movie 2: Ultrafast Selected Area Electron Diffraction at 4 GHz RF excitation.

Supplementary Movie 3: Ultrafast Bright Field Transmission Electron Microscopy movie at 1 MHz electrical excitation with a pulse width of 90 ns.

Supplementary Movie 4: Ultrafast Bright Field Transmission Electron Microscopy images difference movie at 1 MHz electrical excitation with a pulse width of 90 ns using t=-70 ns as the reference.

Supplementary Movie 5: Ultrafast Bright Field Transmission Electron Microscopy movie at 1 MHz electrical excitation with a pulse width of 150 ns.

Supplementary Movie 6: Ultrafast Bright Field Transmission Electron Microscopy movie at 1MHz electrical excitation with a pulse width of 150 ns using t=-100ns as the reference.

Supplementary Movie 7: Ultrafast Selected Area Electron Diffraction movie at 1 MHz electrical excitation with a probe position at t=-70 ns and varying pulse width.

Supplementary Movie 8: Continuous Bright Field Transmission Electron Microscopy movie with RF excitation at 4 GHz showing the sample drift while changing the phase shifter.

Supplementary Movie 9: Mott Resistor Network simulation of the VO_2 synaptic device over one voltage cycle.
